# Osteoporosis in Childhood Cancer Survivors: Physiopathology, Prevention, Therapy and Future Perspectives

**DOI:** 10.3390/cancers14184349

**Published:** 2022-09-06

**Authors:** Francesca Rossi, Chiara Tortora, Marco Paoletta, Maria Maddalena Marrapodi, Maura Argenziano, Alessandra Di Paola, Elvira Pota, Daniela Di Pinto, Martina Di Martino, Giovanni Iolascon

**Affiliations:** 1 Department of Woman, Child and General and Specialist Surgery, University of Campania “Luigi Vanvitelli”, Via L. De Crecchio 4, 80138 Napoli, Italy; 2 Department of Medical and Surgical Specialties and Dentistry, University of Campania “Luigi Vanvitelli”, 80138 Naples, Italy

**Keywords:** childhood cancer survivors, osteoporosis, bone mineral density

## Abstract

**Simple Summary:**

Anti-cancer treatments induced an increase in the childhood cancer survival rate. However, they are responsible for several long-term side effects in childhood cancer survivors, including osteoporosis. Cancer itself, a sedentary lifestyle, and an unhealthy diet might adversely affect bone health. Early identification and adequate management of bone fragility in childhood cancer survivors could be useful to prevent osteoporosis onset and consequently fragility fractures.

**Abstract:**

The improvement of chemotherapy, radiotherapy, and surgical interventions, together with hematopoietic stem cell transplantation, increased childhood cancer survival rate in the last decades, reaching 80% in Europe. Nevertheless, anti-cancer treatments are mainly responsible for the onset of long-term side effects in childhood cancer survivors (CCS), including alterations of the endocrine system function and activity. In particular, the most frequent dysfunction in CCS is a metabolic bone disorder characterized by low bone mineral density (BMD) with increased skeletal fragility. BMD loss is also a consequence of a sedentary lifestyle, malnutrition, and cancer itself could affect BMD, thus inducing osteopenia and osteoporosis. In this paper, we provide an overview of possible causes of bone impairment in CCS in order to propose management strategies for early identification and treatment of skeletal fragility in this population.

## 1. Introduction

During the last decades, the childhood cancer survival rate has considerably increased, reaching 80% in Europe [1,2] and increasing the number of childhood cancer survivors (CCS). This amelioration in survival rate is due to chemotherapy, radiotherapy, surgical interventions’ improvement, and hematopoietic stem cell transplantation (HSCT) [1]. However, cancer treatments are responsible for the onset of long-term adverse effects. The main long-term adverse consequence is the alteration of the endocrine system’s function and activity, which affects about 20–50% of CCS [3]. In CCS, there have been reports of obesity and metabolic syndrome development, impairment of the hypothalamic–pituitary axis, fertility, and bone metabolism, with osteopenia onset and, consequently, an increase in fracture risk [3,4,5,6,7,8,9]. Radiotherapy and chemotherapy are the main causes of the alteration of physiologic cellular and tissue functions, determining an increase in inflammatory processes, senescent cells number, DNA mutations, and an accumulation of reactive oxygen species (ROS) [10,11]. All these factors are responsible for the development of low-grade chronic inflammation. Inflammation causes a further increase in ROS production and of reactive nitrogen species (RNS) which leads to the release of cytokines and other soluble factors and to the stimulation and activation of immune cells [12]. This condition of low-grade chronic inflammation is named “inflammaging” [13] and could be related to the onset of several disorders in CCS [14]. The skeletal sequelae are among the most frequently described complications, affecting 20–50% of subjects [8]. It is widely accepted that cancer therapies influence bone mineral density (BMD) inducing endocrine alterations such as gonadal dysfunction and growth hormone (GH) deficiency [15]. These treatments can also directly affect bone cells. Moreover, additional factors, such as nutritional deficiency and insufficiency of physical activity, have a crucial impact on bone health. It should be underlined that genetic susceptibility may also play an important role in the BMD loss and fragility fracture occurrence in cancer-treated children. A recent genome-wide analysis of BMD in acute lymphoblastic leukemia (ALL) CCS identified complex genetic variants (epistatic interactions), including novel single-nucleotide polymorphisms (SNPs) that potentially modify the effects of specific cancer therapies on BMD [6].

This review provides an overview of the physiopathology of OP in CCS, prevention, therapy, and possible future perspectives to counteract or prevent clinically relevant bone complications and their consequences.

## 2. Bone Metabolism and Physiopathology of Osteoporosis

Bone is a dynamic tissue, whose homeostasis is maintained by a delicate balance between osteoclast and osteoblast activity. Osteoporosis (OP) is the principal bone disease worldwide considering that more than 200 million people suffer from OP. The main pathogenetic factor of OP is the unequal bone remodeling in which osteoclast-mediated bone resorption overcomes osteoblast-mediated bone formation. Several mechanisms are involved in bone formation and bone resorption. Among them, inflammation may contribute to the development of OP [16,17]. During inflammation, the increase in pro-inflammatory mediators including macrophage colony-stimulating factor (M-CSF) that induces the differentiation of monocytes into osteoclasts and receptor activator of nuclear factor-B ligand (RANKL) that acts as an activator of osteoclast-mediated bone resorption [18]. OP can be considered a systemic disease characterized by low bone mass, microstructure alteration of bone tissues, bone fragility, and risk of fractures [19]. OP is classified as “primary” when it occurs in the absence of an underlying disease and as ‘secondary’ when it is due to an underlying disease [19]. Peak bone mass (PBM) is the maximum amount of bone gained at the end of growth. Even though its exact timing is still disputed, it is well documented that the most considerable bone acquisition (almost 94%) occurs during the first 20 years of life [20] with a plateau at the age of 16 [21,22]. Bone mass accumulation is influenced by several factors, such as genetics, ethnicity, calcium and vitamin D intake, physical activity, disease, and drugs adversely affecting bone health [23]. Individuals who do not reach the optimal PBM in puberty, as expected, are at higher risk of OP in adulthood [22]. Several factors, such as increasing age, female sex, prolonged immobility, lack of nutrition, postmenopausal estrogen decrease, glucocorticoid treatments, iron overload, and chemotherapies contribute to the development of OP [24,25,26,27,28,29]. The glucocorticoid-induced OP is a common type of secondary OP [24]. Glucocorticoids, used for inflammatory and autoimmune disease therapy, affect bone cell activity causing a bone mass reduction in 30–50% of treated patients [25,27,28]. Studies demonstrated that chronic glucocorticoid therapy is strongly associated with high susceptibility to fractures [30]. Prednisolone induces osteoblasts and osteocytes apoptosis leading to a reduction in bone formation [30]. It is well known that iron is an important risk factor for OP [31,32]. Iron overload can influence bone formation and remodeling affecting bone microarchitecture and inducing bone loss [33,34]. Generally, iron overload is a consequence of chronic blood transfusions that are mandatory in several diseases such as beta-thalassemia major (TM), hereditary hemochromatosis, and sickle cell anemia [33]. However, a lot of studies reported a strong correlation between iron and inflammation. Proinflammatory cytokines, such as tumor necrosis factor-α, interleukin (IL)-1, IL-6, IL-7, and IL-17, increase the ratio of receptor activator of nuclear factor-κB ligand (RANK-L)/Osteoprotegerin (OPG), which promotes bone resorption. The main factor responsible for the altered iron metabolism is hepcidin, which acts by binding to the iron transporter ferroportin 1 (FPN-1), inducing its internalization and degradation [35].

Cancer-induced OP can derive from the primary disease itself [36]. In this case, the bone alteration can be related to circulating hormones and cytokines produced by the tumor. It is known that patients with primary bone tumors, including osteosarcoma (OS) and Ewing sarcoma, may have bone alteration in the affected sites [1]. Alternatively, cancer-induced OP can derive from the therapies administered to treat the primary condition. Radiotherapy, chemotherapy, surgery, and/or HSCT are known to induce bone loss [5]. Corticosteroids inhibit new bone formation by reducing osteoblastic activity, including osteocalcin production, and by directly improving osteoclastic bone resorption. Moreover, Corticosteroids stop 1α-hydroxylation of vitamin D and induce an impaired intestinal absorption of calcium, reducing muscle strength [5]. Hematopoietic stem cell transplantation (HSCT) also affects bone. Some studies reported that at 6 months after HSCT, nearly 50% of patients showed osteopenia at the femoral neck or lumbar spine. Almost one-third of allogeneic HSCT patients in childhood had reduced bone mineral density (BMD) before reaching adulthood, with a high prevalence of asymptomatic vertebral compression fractures [7]. Other long-term sequelae of cancer treatment, such as gonadal dysfunction, growth hormone deficiency, and altered body composition, have been shown to influence bone remodeling [5]. Patients with ovary or testis tumors are at risk of the onset of primary hypogonadism, affecting BMD. Alkylating agents, such as cyclophosphamide and ifosfamide, also cause primary hypogonadism and result in BMD deficits. Estrogen and androgens influence the growth and maintenance of bone. Estrogen has a role in attaining peak bone mass (PBM) in both sexes. Androgens enlarge the cross-sectional area of long bones and increase mechanical strength [7]. GH and insulin-like growth factor I are important for maintaining bone mass because both independently contribute to bone remodeling and apposition [1].

## 3. Physiopathology of Osteoporosis in CCS: General Risk Factors

Childhood cancer survivors (CCS) are at increased risk of reduced BMD. It is known that CCS fail to attain peak bone mass (PBM) because of cancer itself and the related conditions (e.g., cancer treatments) [37], thus showing a high prevalence of low BMD or even OP in adulthood, with consequent higher risk of bone fragility than the general population [7,38,39]. The mechanism underlying this deficit is multifactorial and firstly depends on the cancer type and the administered therapeutic regimen [40].

In the literature, the evidence on OP-predisposing factors in CCS is controversial [9], but certainly, the lifestyle, the occurrence of endocrine complications, the age at diagnosis, and the genetic predisposition are also recognized as important risk factors [41]. Harmonics and unique clinical practice surveillance guidelines should be implemented to prevent bone fragility in CCS.

Despite nutrition and physical activity being the major determinants of PBM [42], most pediatric cancer survivors do not reach recommended dietary allowances (RDSs) for daily intakes of micro and macronutrients [43] as well as experiencing significant difficulty in engaging in physical activity [44]. Moreover, cancer therapy normally exposes CCS to high risk of long-term metabolic complications [3]. For example, children treated for ALL using cranial irradiation are more predisposed to obesity and related metabolic syndromes [45]. The common alterations in both CCS and cancer patients during therapy are in the metabolism of calcium, vitamin D, and magnesium [39], key micronutrients for bone growth [46]. It has been reported that the 25% of pediatric cancer patients show deficiency of vitamin D [47] and this condition increases the risk of low BMD by more than 3-fold [48]. Vitamin D influences bone mass [49], by modulating the differentiation of bone cells and bone mineralization [50]. Moreover, in 2019, Delvin et al. surprisingly reported that there are no differences in the prevalence of vitamin D deficiency in ALL survivors in comparison to the general population of Canada [51]. Low dietary intake of nutrients necessary for proper growth in CCS is principally due to nausea and/or vomiting caused by oncologic therapy [52], but also to a frequent rejection that patients manifest for healthy foods, preferring higher fat foods [53]. CCS are often unaware of their long-term risks and most of them do not receive appropriate risk-based medical care [54]. Risk factors of chronic nutritional and metabolic alterations could be reduced by prevention interventions, by introducing routine nutritional evaluation in the patients’ follow-up panel, and then eating a low glycemic index/high protein diet, reducing the salt dosage, and increasing the intake of fruit and vegetable [3,55].

CCS are unable to perform adequate physical activity, principally because of long-term hospitalization or poor functional recovery after a surgical intervention, or poor patient compliance [8]. Immobilization enhances bone resorption and negatively influences bone mass acquisition [56]. Moreover, a sedentary lifestyle results in both cardiac deconditioning and skeletal muscle atrophy [52]. All these aspects are responsible for a condition of “disuse osteoporosis” [8]. There is a vicious circle in which the patient forced into immobilization by cancer itself will then develop a physical and psychological state that makes it difficult to resume proper levels of activity. Several authors have already reported that improvement in physical activity is associated with higher BMD Z-scores in survivors of different kind of tumors [41,57]. This evidence has been further consolidated with the prospective trial PASTEC [58]. The authors performed a randomized study on 22 oncology patients aged 6–18 years old and observed a positive impact of physical activity on anxiety, emotional state, and motor capabilities, thus encouraging the introduction of motivational interventions in CCS aimed at improving physical activity levels. Exercise and physical activity must be tailored to personal health conditions and preference. For example, in a group of 120 CCS aged 8–18 years from South Korea, it was observed that the most preferred activities were soccer, basketball, badminton, and dance with important beneficial effects, especially on mood [59,60].

Even though the evidence is currently very limited, genetics also seems to influence bone density in children with cancer. Te Winkel et al. observed that variations in genes involved in folate metabolism negatively affect bone mass density in ALL patients at diagnosis [61], as well as the finding that a polymorphism of corticotrophin-releasing hormone receptor-1 gene is related to low bone density in male ALL survivors [6]. Furthermore, in 2020, Im and collaborators performed genome-wide association studies of fracture risk after cancer diagnosis in patients from the Childhood Cancer Survivor Study (CCSS), identifying a genetic locus (HAGHL, 16p13.3) for fracture risk [6]. A few years earlier, it was demonstrated by multivariate analyses that CCS carrying SNPs in the ESR1 (estrogen receptor type 1) or LRP5 (low-density lipoprotein receptor) genes had an impairment in bone mass at an early adult age [62]. Given these considerations, developing genetic screening strategies could be useful to improve the prediction of bone fracture risk and prevent it in CCS.

## 4. The Contribution of Cancer Therapies to Bone Mass Loss in CCS

Cancer therapies including chemotherapy (particularly glucocorticoid (GC) and methotrexate), radiotherapy, and HSCT can negatively influence the achievement of the PBM in CCS. The prevalence of low BMD among CCS is about 9–51% [63]. High-dose GC, prednisone, and methotrexate, commonly used for childhood leukemia treatment, significantly induce a decrease in BMD by inhibiting osteoblastic proliferation and activity [64]. Radiotherapy can induce damage to the hypothalamic–pituitary axis that results in growth hormone (GH) deficiency and hypogonadism, which prevent bone growth and mineral acquisition [65]. Low BMD in childhood and adolescence can lead to OP in adults along with fragility fractures and other skeletal adverse events, such as bone pain and bone deformity resulting in disability and poor quality of life [66]. Bone mineral density reduction has been reported for several pediatric cancers [44,67,68] such as ALL [69,70], lymphomas, neuroblastoma [71], and as a complication after HSCT. Leukemic cell proliferation in the bone marrow and cytokine-mediated osteoclast activity are suggested as contributing factors to low BMD [70]. Patients with primary bone tumors, including OS and Ewing sarcoma, have low BMD in the affected sites [72,73,74]. Bloomhardt et al. revealed a significantly increased risk of fragility fracture with low lumbar spine BMD in survivors of childhood leukemia/lymphoma 2 years off therapy [4]. Less is known regarding the frequency of OP in patients with solid tumors [75]. Studies reported an increased risk of low BMD during and immediately after the cessation of childhood cancer treatments but still little is known about the long-term effects [76,77]. Probably, osteoclast hyperactivation is sustained by chronic low-grade inflammation [13]. Indeed, exposure to oncogenic treatments leads to inflammation by activating an immune response which could persist, even after a long time, after irradiation, chemotherapy, or HSCT [78]. Studies suggest the association between increased pro-inflammatory cytokines activity and bone loss. Indeed, cytokines can stimulate osteoclasts inducing their overactivation and consequently bone loss [14]. In this section, we address the contribution of the different cancer therapies to bone mass loss in CCS.

### 4.1. Chemotherapy

**Figure 1:**
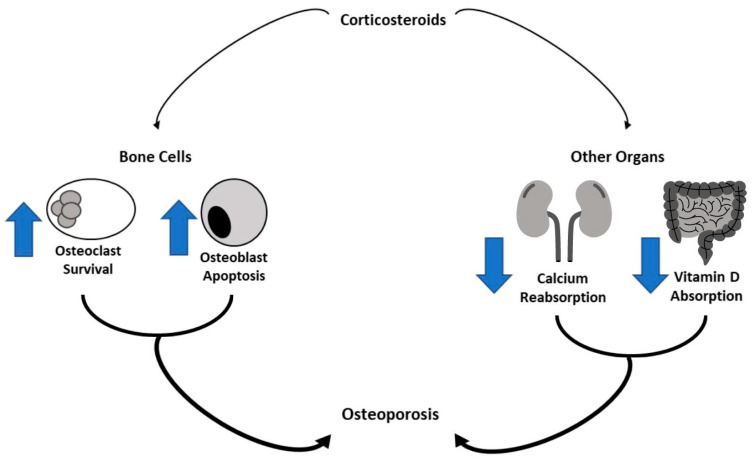
Corticosteroids effect bone metabolism. Corticosteroids have inhibitory effects on bone cells by increasing osteoclast survival and osteoblast apoptosis. They also reduce the absorption of vitamin D in the intestinal tract and calcium reabsorption in the renal tubule, determining bone reabsorption.

The effects of chemotherapy on bone metabolism have been widely studied [44,64,65,79]. The chemotherapeutic agents most frequently used to treat pediatric cancer are corticosteroids (CS) and methotrexate. CS are used in the treatment of most childhood tumors such as ALL and Hodgkin and non-Hodgkin lymphoma for increasing anticancer therapy and to prevent several side effects such as nausea and allergy [8,80]. Moreover, CS have a central role in the prophylaxis for graft versus host disease (GVHD) in patients undergoing hematopoietic stem cell transplant (HSCT) [81]. It is amply known that CS have inhibitory effects on bone cells by increasing the survival of osteoclasts and by affecting osteoblast activity and differentiation [82,83]. In addition to a direct effect, CS induce bone reabsorption also acting on other organs (Figure 1). They have catabolic effects on muscle inducing muscle weakness with reduced mechanical loading on the bone that increases the fracture risk. CS reduce the intestinal absorption of calcium and vitamin D and the calcium reabsorption in the renal tubule. Moreover, CS reduce gonadotropin secretion inhibiting sex steroids secretion. CS have a more major effect on trabecular bone (more metabolically active) than cortical bone, therefore fragility fractures due to CS occur mainly in vertebrae [84]. The contribution of these drugs to reducing bone mass closely depends on the dose itself and the time of exposure [85]. Several studies demonstrated the negative effects of CS on CCS bone mass. Frieze et al. suggested a higher risk of OP and fracture along with an increase in CS dose and duration [86]. The bone loss occurs rapidly, within a few days of GC exposure and it is similar in the lumbar spine and femoral neck [87]. A longer duration of GC use was related to an increased risk of fracture [15]. In a cohort of 245 long-term childhood ALL survivors, Fiscaletti et al. demonstrated a significant prevalence of vertebral deformities and identified male sex, cumulative CS dose, and back pain as predictors of vertebral deformity underling the importance of bone health surveillance in ALL survivors [88]. Regarding methotrexate, it has been significantly associated with reduced BMD in children treated for childhood cancer [89]. It exerts a cytotoxic effect on osteoblasts inducing impaired bone formation and reduced bone volume. Moreover, this drug stimulates osteoclast recruitment. In detail, bone damage induced by methotrexate is dose-dependent and it is linked to molecular alteration of the expression of Runx-2 and OSTERIX, transcription factors involved in osteoblast differentiation [70]. Higher cumulative doses of methotrexate have been associated with a greater incidence of bone loss and with failure to recover BMD after chemotherapy completion [73].

### 4.2. Radiotherapy

Radiotherapy is commonly used as therapy for several childhood cancers, including leukemia, lymphoma, brain tumors, sarcomas, neuroblastoma, and nephroblastoma [90]. Studies suggest that local and total body irradiation may affect BMD directly by affecting the bone marrow stroma [8]. Moreover, it has been suggested that radiotherapy can induce bone loss by activating osteoclasts [91]. The bone damage induced by radiotherapy is closely related to its effects on the hypothalamic–pituitary axis that result in growth hormone (GH) deficiency and hypogonadism which impair bone growth and mineral apposition [92].

An increased risk of BMD loss among survivors with GH deficiency has been reported [93]. However, a study of ALL survivors treated with five years of GH replacement therapy reported no beneficial effect for BMD [94]. The effects induced by radiotherapy depend on the radiation source, cumulative dose, volume, the fraction of radiation, and sex and age at the time of treatment [95]. Young age, higher radiation dose, and pretransplant radiation can increase the risk of GH deficiency. A study on survivors of various cancer diagnoses demonstrated a 3.6-fold increased risk of BMD loss among survivors exposed to cranial radiation compared to those not exposed to radiation [96]. The negative effects of radiotherapy on BMD have been specially reported in children with brain tumors. Patients with craniopharyngioma, germinoma or low-grade glioma develop growth hormone (GH) deficiency that consequently can affect BMD [97,98].

### 4.3. Hematopoietic Stem-Cell Transplantation

Autologous and allogeneic HSCT is the treatment of choice for patients with some malignant and non-malignant hematological diseases. Advances in transplantation techniques have significantly increased the number of long-term HSCT survivors who are at risk for developing several complications including reduced bone density [99]. This latter condition is common among HCT survivors with an incidence of 50% to 75% after allogeneic HSCT [100,101,102] and 20–65% after autologous HSCT [103]. Bone loss commonly occurs within 3 to 6 months after transplantation [104]. Survivors with reduced BMD are at increased risk of post-transplantation fragility fractures that occur spontaneously or after a trauma. This risk is higher after autologous HSCT and it is well documented in patients with multiple myeloma [105,106]. HSCT indirectly affects bone health by influencing the duration of hospitalization and physical inactivity [107]. Post-transplantation risk factors for bone loss include secondary hyperparathyroidism, chronic kidney disease, renal wasting of calcium or magnesium, impaired liver function, and granulocyte colony-stimulating factor (G-CSF) therapy [81]. Moreover, the iron overload that commonly occurs after HSCT could further increase the risk of OP [108]. A large retrospective study demonstrated that among 5000 HSCT adults, 87% had received high-dose GC therapy and 25% had a pre-HCT fracture [109] suggesting that GC exposure is one of the strongest risk factors for post-HCT bone involvement [101]. Therefore, the identification of risk factors for bone loss is a key strategy to provide an effective prevention approach for improving or maintaining bone health before HSCT.

## 5. Bone Loss in CCS with a Different Cancer Diagnosis

Other than cancer treatments, several causes are responsible for bone metabolism damage, particularly decreased physical activity and nutritional deficiencies (Table 1) [52,110,111]. It has been demonstrated that BMD could be also directly influenced by the pathogenic mechanism of cancer [8,52]. Indeed, cancer itself inhibits long-term CCS from achieving an optimal PBM [39]. In particular, low BMD was observed in patients with ALL at diagnosis [69,79] and in patients with neuroblastoma [71]. Indeed, the proliferation of leukemic cells in the bone marrow and cytokines released by active osteoclasts are considered the main contributors to low BMD [79]. This finding is also observed in patients with OS and Ewing’s Sarcoma [73,74]. The impairment of bone mass acquisition during childhood could induce the onset of OP and fragility fractures in adult years [7,39,52].

BMD reduction is observed in children survivors of leukemia, lymphoma, brain tumors, solid tumors, and those who undergo HSCT [7].

### 5.1. ALL and Lymphoma

ALL is the most common hematologic malignancy in childhood, indeed, it represents 25% of all childhood cancers [112]. At the time of diagnosis, 10–20% of children with ALL show a strong reduction in BMD at the lumbar spine [7,39,70] and VF in 16% of patients at diagnosis [113]. It has been reported that in ALL children there is a 6-fold increased risk in developing fragility fractures compared to healthy subjects [4]. Indeed, in these patients, the radiography reveals lytic and sclerotic lesions, and periosteal elevations [52]. In ALL children, BMD could be influenced directly by both the leukemic process and cancer treatment [52], which usually consists of corticosteroids and methotrexate administration [39].

ALL children are more prone to the development of several other skeletal complications, among them fracture, osteopenia, osteonecrosis, bone deformation, OP, and bone pain during or after treatment [114]. ALL therapy contributes to the significant impairment of BMD, mainly in the early phases in which treatments are more intensified [39]. During treatment, BMD shows a significant reduction compared to the levels measured at diagnosis [52,79]. This condition results in a higher risk of fracture, in about 39% of ALL children in treatment [39]. There are several studies reporting evidence of BMD variation in ALL patients after treatment. For example, it has been demonstrated that after 15.9 years ALL patients show a low BMD in 21% of cases, compared to 5% of the general population of the same age and sex [84]. Conversely, other studies reported that ALL patients recover a normal BMD after stopping treatment [79,115,116]. For example, subjects enrolled within 6 months after the end of therapy showed a reduction in cortical volumetric BMD at QCT whereas subjects who were more than 6 months post-chemotherapy recovered cortical volumetric BMD, demonstrating acquisition of cortical bone [117]. These data have been proven in several other studies with an increase in BMD in the years immediately following the end of therapy [70,118,119].

Lymphoma represents the third most common childhood cancer in Great Britain and northern Europe (10%) [120]. The main lymphoma subgroups are represented by Hodgkin lymphoma and non-Hodgkin lymphoma [120]. It has also been demonstrated that 41% of Hodgkin and 50% of non-Hodgkin lymphoma CCS will lose BMD at the lumbar spine [121,122]. Moreover, the administration of prednisone, vincristine, procarbazine, and mechlorethamine induces a reduction in height together with an increase in body mass index (BMI) in men, while in women it determines a reduction in BMD [123]. It has also been reported that a high dose compared to a low dose of CS significantly increases the risk of osteopenia in this population [122].

### 5.2. Brain Tumors and Neuroblastoma

Brain tumors are the most common childhood solid tumors [124]. Neuroblastoma is the most frequent in pediatric age and is responsible for 15% of childhood cancer-related mortality [125]. In brain tumors, CCS show a significant reduction in BMD as well as a significant incidence of OP as common consequences of cancer therapy [39,98,126]. Several factors affect BMD in brain tumor CCS, in particular, GH deficiency and craniospinal high doses of CS and alkylating agent administration [98,126]. Kang and collaborators observed both OP (25%) and osteopenia (42.9%) in patients with germ cell tumors at 10.9-year follow-up [127]. It has also been demonstrated that BMD loss at the femoral neck and lumbar spine occurs in 47.3% of brain tumor CCS [39]. Accordingly, other studies demonstrated that brain tumors CCS are more prone to the development of early OP and fractures when associated with the risk factor GHD [128,129].

Moreover, relevant bone involvement is also observed in neuroblastoma CCS treated with high doses of therapy and HSCT [8,130]. Indeed, although these treatments improve survival rates, they could also be responsible for inducing an alteration in the growing skeleton, such as limb length discrepancy, short stature, osteonecrosis, scoliosis, and osteochondromas [8,131].

### 5.3. OS and Ewing’s Sarcoma

OS is the most prevalent malignant bone tumor in childhood characterized by a high rate of metastasis [132,133,134,135,136]. Ewing’s sarcoma is the second most frequent and highly aggressive bone tumor in childhood [137]. Although neoadjuvant chemotherapy has increased the survival rate of OS and Ewing’s sarcoma patients [8,138], CCS show a reduction in PBM along with the development of osteopenia/OP onset, and the onset of fragility fractures [72,139,140]. Holzer and collaborators reported that in 48 OS long-term survivors, 10 of them show OP, while 21 were osteopenic [72]. Other studies also reported a decrease in BMD at the lumbar spine and femur neck in OS and Ewing’s Sarcoma CCS after neoadjuvant chemotherapy [73,141]. It has also been demonstrated that lean mass reduction, male sex, and young age at diagnosis could be considered risk factors for OP in OS CCS [139].

### 5.4. Wilm’s Tumor

Wilm’s tumor is the most common childhood renal tumor, representing 7% of all childhood cancers [142]. BMD loss was also observed in two different small series of Wilm’s tumor CCS after therapy [143]. In particular, there have been reports of high osteopenia rate (27%) in CCS of Wilm’s tumor along with an imbalance of bone turnover, characterized by excessive bone resorption [143].

## 6. Therapy and Prevention

Management of children affected by metabolic bone disease needs a multidimensional approach including pharmacological and non-pharmacological therapy. Considering the high bone turnover caused by cancer disease and related therapies, the administration of a proper therapy must be carefully evaluated for reducing bone loss and the onset of skeletal-related events (SRE). CCS often present an aberrant Wnt/β-catenin signaling pathway responsible for both pro-tumor effect and pro-resorptive action on bone mass [144]. Considering this aspect, therapeutical interventions could be useful in preventing low BMD in CCS. Bisphosphonates (BPs) are the main therapeutic approach for treating OP, even in children [145], since they act as antiresorptive agents inhibiting osteoclast activity. They are recommended and widely used for pediatric skeletal diseases, such as osteogenesis imperfecta [146] and Paget’s disease [147]. Since chronic oral administration could damage gastric mucosa by reducing nitric oxide synthase expression, in children the parenteral administration could be preferred, considering also the low rate of adverse events, such as hypocalcemia ad hypophosphatemia [148]. CCS commonly assume several drugs that could interact with BPs, including anti-convulsants, gabapentinoids and muscle relaxers. Among BPs, Zoledronate (ZOL), Pamidronate (PAM) and Neridronate (NRD) are the most-used anti-osteoporotic agents in children. ZOL is used in children affected by cancer who are disease free for a prolonged time and also in cases of bone metastases [149], as adjuvant chemotherapy to reduce skeletal events [150]. Recently, Liu et al. observed in their study a reduction in osteolysis with combined use of Cisplatin and ZOL (CisZol) in OS patients, due to a strong inhibition of osteoclastic differentiation [150]. PAM also showed positive effects on OS pediatric patients, increasing lumbar spine BMD with little relevant adverse effects, such as acute-phase reaction and hypocalcemia [151]. The therapeutic schedule consists of 1 mg/kg administered over 3 consecutive days, every 4 months [152]. Beneficial effects were found also in ALL and non-Hodgkin’s lymphoma with an increase in lumbar spine BMD. However, the main important limit in using PAM is the need for several more hospital accesses per year than ZOL. Therefore, considering the comparable efficacy, PAM therapy seems to be more expensive than ZOL [153]. NRD, an amino-bisphosphonate available in both intramuscular and intravenous formulations, is licensed for the treatment of children with OI and PBD [154]. This agent provides a significant biochemical remission in PDB and an increase in BMD at the lumbar spine up to about 50% at 3 years in patients with OI. Moreover, considering the good safety profile of NRD in the pediatric population, it could represent an effective alternative therapeutic approach for CCS with low bone mineral density.

Denosumab (Dmab) is a human monoclonal antibody targeting RANKL that significantly reduces the risk of vertebral and non-vertebral fracture in osteoporotic patients [155]. Since there are no proper studies on its use on adolescents and children, Dmab is still off-label for pediatric patients. Only a few studies have been conducted on adolescents with low BMD showing several limitations [156]. In particular, some issues in identifying the correct dosage according to weight and body surface, rebound phenomena at suspension, increased bone turnover markers, and critical levels of hypocalcemia for cardiac repolarization [157]. Moreover, a recent study by Punzo et al. discourages the use of Dmab in OS pediatric patients, since it is not able to contain tumoral progression or ameliorate the effects of the already-used drug, Doxorubicin [134].

In CCS, the management of bone involvement should include multidimensional intervention: pharmacological treatments, nutritional approaches, and exercise programs. Patients who survived childhood cancer usually fail to reach recommended daily allowances [144,158,159]. The PETALE study showed that more than 60% of children and young adults who survived ALL consume insufficient amounts of fruit, vegetables, and fiber [160]. These patients mainly use ultra-processed foods and high-fat foods sustaining low-grade systemic inflammation and reducing the intake of nutrients for bone metabolism [154]. Activated T cells are major stimulators of osteoclastogenesis by TNF-α, IL-1, and RANKL, powerful stimulators of bone resorption and inhibitors of bone formation.

Proteins play a pivotal role in bone metabolism in pediatric patients with low mineral density, indeed, one-third of bone mass is made up of proteins. Inadequate protein intake during growth could severely impair bone development causing delayed skeletal growth and reduced bone mass. On the other hand, protein intake must be appropriately balanced since the use of chemotherapy, such as methotrexate, cyclophosphamide or anthracyclines, could lead to late-onset renal failure in CCS [153,161]. In these cases, protein intake should be carefully scheduled avoiding high-dose intake with the risk of intraglomerular pressure and glomerular hyperfiltration.

In cancer patients, a low serum level of calcium and vitamin D is commonly found. In particular, vitamin D deficiency is mainly related to lifestyle changes. Indeed, children with cancer and CCS are advised to avoid sun exposure during chemotherapy due to the risk of photosensitivity or secondary skin neoplasm reducing the activation of vitamin D [153]. Beyond lifestyle, the assumption of pharmacological therapy could influence calcium and vitamin D metabolism. Corticosteroids, widely used in children with lymphoma, OS, and leukemia, may decrease intestinal absorption of vitamin D and its activation inhibiting vitamin D 1α-hydroxylation. Therefore, monitoring 25 OH-vitamin D and calcium levels in CCS is mandatory in this population. Normal levels of vitamin D are over 30 ng/mL, and levels between 20 and 30 ng/mL and <20 ng/mL are considered deficient and insufficient, respectively [162]. In particular, in CCS a vitamin D supplementation of at least 400 units per day is suggested for recovering or maintaining adequate serum levels [163]. On the other hand, calcium supplementation in CCS is not well investigated. Calcium is mainly supplemented as carbonate or citrate formulation, even though calcium citrate could be associated with less severe gastrointestinal adverse events, such as constipation and flatulence [164].

Among the most innovative frontiers in the research, nutraceuticals, namely “food (or parts of a food) seems to provide health benefits, including the prevention and/or treatment of diseases” [165]. These compounds could have anti-inflammatory and antioxidant properties and a positive effect on bone metabolism in the chemotherapy-induced OP in children [166]. For example, in a recent animal study, it has been demonstrated that resveratrol reduces cellular oxidative stress and, as a consequence, the expression of osteoclast-specific factors, such as TRAP-5b and RANKL [167]. Furthermore, Lee et al. demonstrated that resveratrol during methotrexate treatment reduces fatty replacement of bone marrow [168]. Another promising compound is genistein which shows direct effects in the chemotherapy-induced OP in animal models. It seems to inhibit osteoclast proliferation in the bone marrow of rats treated with methotrexate, preventing bone resorption [169]. Similarly, icariina reduces bone loss induced by chemotherapeutic agents, through the activation of the Wnt/catenin signal. However, these effects are not yet demonstrated in humans and further studies are certainly needed [170].

Physical activity plays a crucial role in stimulating bone metabolism, restoring muscle and skeletal function, and/or maintaining a state of well-being [171]. Physical exercise increases Wnt/βchain signal that stimulates osteogenesis and bone neoformation [172]. Mechanical stress also stimulates osteoprotegerin release from osteoblasts. This protein exerts its effects as a decoy receptor of RANKL inhibiting its binding to the RANK expressed by osteoclasts, thus preventing bone resorption [173]. Only a few studies investigated the effects of physical exercise on CCS. In 2020 Zürcher and collaborators confirmed the efficacy and safety of high-impact exercise, for example jumping and fast running, in 161 CCS [174]. This study is in contrast with the previous evidence in the literature. A study performed in 2009 evidenced no significant differences in BMD at the lumbar spine in a patient with ALL, although physical exercise maintains and improves functional outcomes in this population [175]. Accordingly, in 2016, Braam and collaborators did not highlight a statistically significant difference in BMD in CCS performing exercise in terms of BMD [176]. Therefore, although the benefits of physical activity for bone metabolism are well known, further studies are needed to identify in detail the type of exercise intensity, sessions per week, and any precautions.

## 7. Conclusions and Future Perspectives

**Figure 2:**
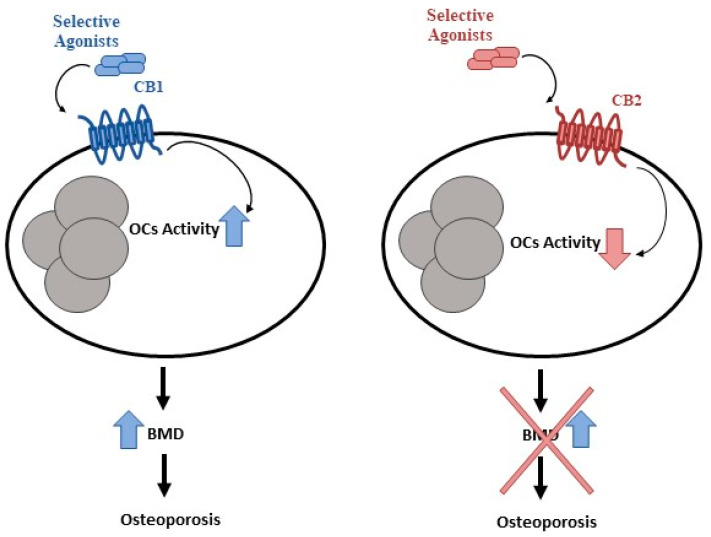
CB1 and CB2 in bone metabolism. Cannabinoids and their receptors, CB1 and CB2, play a key role in bone metabolism. CB1 activation stimulates osteoclast activity, whereas CB2 activation inhibits osteoclast activity.

Cancer therapies expose patients to risk of developing long-term sequelae. Bone health is frequently involved and often accompanied by bone pain, growth impairment, and fragility fractures which may cause disability and poor quality of life [66]. The assessment of this aspect in CCS during therapy and after cessation of treatment as well as a long-term follow-up is necessary to maintain bone health in these patients. Considering the long-term skeletal conditions in CCS, and the lack of treatments for reducing this damage, it is mandatory to recognize the underlying mechanisms in order to propose and analyze new treatments. Moreover, early identification of CCS with high fracture risk could reduce morbidity and the cost to the national health system. It is widely known that cannabinoids and their receptors, CB1 and CB2, play a role in bone metabolism, CB1 activation stimulates osteoclast activity, whereas CB2 activation inhibits osteoclast activity and promotes osteoblast function [177,178,179,180] (Figure 2). Several studies suggested CB2 as a possible marker to identify patients at risk of fractures in different bone loss-related pathologies and as a therapeutic target. Therefore, possible CB2 involvement in CCS-related OP is not excluded. Bone mineral density involvement has been reported after treatment for several pediatric cancers such as OS [141]. Accordingly, an in vitro study reported a strong increase in osteoclast activity in OS patients together with a reduced level of the CB2 receptor. Interestingly, CB2 reduction is more marked when patients are undergoing chemotherapy. Chemotherapy-induced effects on bone resorption are reverted in osteoclasts derived from OS patients in the chemotherapy plus mifamurtide, suggesting a role for this drug as an anti-resorption agent in the chemotherapy-induced OP in children with OS [133]. Several experimental studies demonstrated that chemotherapy with methotrexate (MTX), the most-used drug in childhood oncology, increases bone resorption and induces severe growth dysfunction [181]. Several studies suggested that the supplementation of specific nutraceuticals such as flavonoids, fatty acids, and resveratrol may counteract MTX chemotherapy-induced bone loss [166,168,182]. Flavonoids are effective in protecting bone during cancer chemotherapy [166]. Fatty acids can counteract the inflammatory condition induced by MTX to reduce osteoclastic bone resorption and preserve bone formation in rats [182]. Certainly, more experimental and clinical studies are needed to investigate the interesting potential of these novel therapeutic compounds, evaluating the optimal doses required to prevent chemotherapy-induced bone damage and their mechanisms of action.

Another important aspect is that cancer survivors require long-term health care. They often require management of treatment-related late effects and monitoring for subsequent malignant diseases [183]. Furthermore, cancer survivors are more likely to experience physical and mental health issues, thus they often need a comprehensive and multidisciplinary management [184]. For these reasons, the access to quick and cheap care for these individuals is mandatory. Nevertheless, these subjects are burdened by the high costs of their care, which may negatively influence their quality of life and access to care [185,186]. Financial concerns represent a barrier to accessing medical services among cancer survivors [187]. Studies reported that patients with a cancer history experience issues related to disability, loss of work, and difficulty obtaining health insurance [188,189,190]. However, barriers to accessing care experienced by cancer survivors remain still understudied. Policy makers and stakeholders should raise a greater awareness for the improvement of the diagnostic and therapeutic pathway for these patients.

## Figures and Tables

**Table 1:** Risk factor for osteoporosis in several type of cancers.

Type of Cancer	Risk Factor for Osteoporosis
**Acute Lymphoblastic Leukemia (ALL)**	ALL treatments (corticosteroids and methotrexate)
**Lymphoma**	Lymphoma treatments (prednisone, vincristine, procarbazine, low dose of corticosteroids, and mechlorethamine)
**Brain Tumor**	GH deficiency and craniospinal high doses of corticosteroids and alkylating agent administration
**Neuroblastoma**	High doses of therapy and hematopoietic stem cell transplantation (HSCT)
**Osteosarcoma**	Neoadjuvant chemotherapy, lean mass reduction, male sex, and young age at diagnosis
**Ewing’s Sarcoma**	Neoadjuvant chemotherapy
**Wilm’s Tumor**	Chemotherapy
